# Impact of *IL28B*, *APOH* and *ITPA* Polymorphisms on Efficacy and Safety of TVR- or BOC-Based Triple Therapy in Treatment-Experienced HCV-1 Patients with Compensated Cirrhosis from the ANRS CO20-CUPIC Study

**DOI:** 10.1371/journal.pone.0145105

**Published:** 2015-12-15

**Authors:** Frédégonde About, Tiphaine Oudot-Mellakh, Jonathan Niay, Pascaline Rabiéga, Vincent Pedergnana, Darragh Duffy, Philippe Sultanik, Carole Cagnot, Fabrice Carrat, Patrick Marcellin, Fabien Zoulim, Dominique Larrey, Christophe Hézode, Hélène Fontaine, Jean-Pierre Bronowicki, Stanislas Pol, Matthew L. Albert, Ioannis Theodorou, Aurélie Cobat, Laurent Abel, ANRS CO20-CUPIC study group

**Affiliations:** 1 Laboratory of Human Genetics of Infectious Diseases, Necker Branch, Institut National de la Santé et de la Recherche Médicale (INSERM) U1163, Paris, France; 2 Paris Descartes University, Imagine Institute, Paris, France; 3 Laboratory of Immunity and Infection, Centre d’Immunologie et des Maladies Infectieuses de Paris (CIMI), INSERM U1135, Groupe Hospitalier Pitié Salpétrière, Assistance Publique-Hôpitaux de Paris (AP-HP), Paris, France; 4 Plateforme Génomique Inserm-ANRS, Groupe Hospitalier Pitié Salpétrière, AP-HP, UPMC Université Paris 6, Paris, France; 5 Sorbonne Universités, UPMC Université Paris 06, INSERM, Institut Pierre Louis d’épidémiologie et de Santé Publique (IPLESP UMRS 1136), Paris, France; 6 Wellcome Trust Centre for Human Genetics, University of Oxford, Oxford, United Kingdom; 7 Centre for Human Immunology, Department of Immunology, Institut Pasteur, Paris, France; 8 The Laboratory of Dendritic Cell Biology, Department of Immunology, Institut Pasteur, INSERM U818, Paris, France; 9 Département d'Hépatologie, Hôpital Cochin, AP-HP, Université Paris Descartes, Paris, France; 10 INSERM UMS20, Institut Pasteur, Paris, France; 11 Unit for Basic and Clinical research on Viral Hepatitis, Inserm-ANRS (France REcherche Nord & sud Sida-HIV Hépatites-FRENSH), Paris, France; 12 Service de Santé Publique, Hôpital Saint Antoine, AP-HP, Paris, France; 13 Service d’Hépatologie, Hôpital Beaujon, Clichy, France; 14 Centre de recherche en cancérologie de Lyon (CRCL), INSERM UMR I 1052/CNRS 5286, Lyon cedex 03, France; 15 Université Claude-Bernard Lyon 1, Villeurbanne, France; 16 Hospices civils de Lyon, Hôpital de la Croix-Rousse, service d'hépatologie et de gastroentérologie, Lyon, France; 17 CHU St Eloi Hospital, Liver Unit, Montpellier, France; 18 Department of Hepatology and Gastroenterology, Hôpital Henri Mondor, AP-HP, Université Paris-Est Créteil (UPEC), Créteil, France; 19 Institut Mondor de Recherche Biomédicale (IMRB), INSERM U955, UPEC, Créteil, France; 20 Department of Hepatogastroenterology, INSERM U954, CHU de Nancy, Université de Lorraine, Vandoeuvre-Lès-Nancy, France; 21 St. Giles Laboratory of Human Genetics of Infectious Diseases, Rockefeller Branch, The Rockefeller University, New York, NY, United States of America; National Taiwan University Hospital, TAIWAN

## Abstract

**Background:**

Human genetic factors influence the outcome of pegylated interferon and ribavirin hepatitis C therapy. We explored the role of *IL28B*, *APOH* and *ITPA* SNPs on the outcomes of triple therapy including telaprevir or boceprevir in patients with compensated cirrhosis chronically infected with HCV-1.

**Patients and Methods:**

A total of 256 HCV-1 Caucasian treatment-experienced patients with compensated cirrhosis from the ANRS CO20-CUPIC cohort were genotyped for a total of 10 candidate SNPs in *IL28B* (rs12979860 and rs368234815), *APOH* (rs8178822, rs12944940, rs10048158, rs52797880, rs1801689 and rs1801690) and *ITPA* (rs1127354 and rs7270101). We tested the association of *IL28B* and *APOH* SNPs with sustained virological response and of *ITPA* SNPs with anemia related phenotypes by means of logistic regression assuming an additive genetic model.

**Results:**

None of the six *APOH* SNPs were associated with sustained virological response. The favorable alleles of the *IL28B* SNPs rs12979860 and rs368234815 were associated with sustained virological response (rs12979860: OR = 2.35[1.50–3.70], *P* = 2x10^-4^). Refined analysis showed that the effect of *IL28B* SNPs on sustained virological response was restricted to prior PegIFN/RBV relapse (OR = 3.80[1.82–8.92], *P* = 8x10^-4^). We also confirmed the association between *ITPA* low activity alleles and protection against early hemoglobin decline in triple therapy (*P* = 2x10^-5^).

**Conclusion:**

Our results suggest that the screening of rs12979860 may remain interesting for decision making in prior relapse HCV-1 Caucasian patients with compensated cirrhosis eligible for a telaprevir- or boceprevir-based therapy.

## Introduction

HCV infection is a major public health issue with ~80 million people chronically infected worldwide [1]. Up to 2011, standard of care treatment was based on pegylated interferon and ribavirin (PegIFN/RBV) which leads to viral clearance in ~50% of the patients [2]. Well-established baseline predictors of sustained virological response (SVR) to PegIFN/RBV include viral load, HCV genotype, age, ethnicity, body weight, insulin resistance, steatosis, fibrosis stage, and *IL28B* single nucleotide polymorphism (SNP) rs12979860 [2–5]. A dinucleotide frameshift variant (rs368234815) creating a novel gene encoding *IFN-λ-4*, in strong linkage disequilibrium (LD) with rs12979860, was recently identified as a stronger predictor than rs12979860 of treatment-induced clearance [6]. One of the most common side effects of ribavirin therapy is anemia that mainly appears at the beginning of treatment. Two variants, rs1127354 and rs7270101, from the inosine triphosphate (*ITPA*) gene, encoding a protein that hydrolyses inosine triphosphate, are independent predictors of RBV-induced anemia [7].

Since 2011, first generation direct acting antiviral drugs (DAAs) targeting the HCV NS3/4A protease, such as telaprevir (TVR) and boceprevir (BOC), are available in several countries. These drugs combined with a PegIFN/RBV backbone significantly improved the SVR as compared to PegIFN/RBV alone in both treatment-naive and previous treatment-failure patients with chronic HCV genotype 1 (HCV-1) infection [8,9]. However, side effects such as anemia are more frequent, particularly in patients with cirrhosis [10]. More recently, new IFN-free therapies with second generation DAAs have emerged and provide SVR rates over 90% [11]. However, these therapies are still very expensive and not yet widely used in real life settings. Therefore, triple therapy combining PegIFN/RBV with first generation protease inhibitors (PIs) remains the standard of care for HCV-1 infected patients in most countries. In the present study we aimed to explore the role of *IL28B*, *APOH* and *ITPA* SNPs on the outcomes of triple therapy including telaprevir or boceprevir in patients with compensated cirrhosis chronically infected with HCV-1.

## Patients and Methods

### Study population

The ANRS CO20-CUPIC (Compassionate Use of Protease Inhibitors in viral C Cirrhosis) study is a French multicenter cohort study that enrolled 660 HCV genotype 1 (HCV-1) treatment-experienced cirrhotic patients to assess safety and efficacy of triple therapy with TVR or BOC for difficult to treat patients in real-life settings [10,12]. Briefly, patients with compensated cirrhosis chronically infected with HCV-1, and who failed a prior course of IFN alone or IFN/RBV started a triple combination therapy including PegIFN/RBV and TVR or BOC for a total course of 48 weeks [10]. The choice between TVR and BOC was at the investigator’s discretion. Results showed a substantial benefit of triple therapy in difficult to treat patients with SVR rates of 43–52% but with an increased frequency and severity of side effects [12]. Interestingly, a recent study conducted in 189 patients from the CUPIC cohort identified baseline levels of apolipoprotein H (apoH), encoded by *APOH* gene, as a surrogate marker for SVR to triple therapy [13]. *APOH* polymorphisms have previously been associated with triglyceride levels, which itself is an independent correlate of HCV clearance [14].

Written informed consent was obtained from each patient before enrolment. The study was conducted in accordance with the Declaration of Helsinki and French law for biomedical research and was approved by the “Ile de France IX” Ethics Committee (Créteil, France).

### Outcomes and statistical analysis

In the present study we took advantage of the well characterized CUPIC cohort study to assess the role of candidate SNPs in *IL28B*, *APOH* and *ITPA* on efficacy and safety of TVR- or BOC-based triple therapy. Only Caucasian patients who gave their consent for genetic testing were included (n = 256). Efficacy was assessed by SVR, defined as an undetectable HCV-RNA level 12 weeks after the end of therapy. For safety analysis, we focused on anemia and first considered a broad definition of clinically relevant anemia corresponding to patients with grade 2, 3 or 4 anemia (i.e. Hb<9.5g.dl^-1^) and/or blood transfusion and/or use of erythropoietin (EPO) occurring during the 48 weeks of treatment. We also focused on early significant hemoglobin decline, defined as a decrease of hemoglobin level of at least 3g.dl^-1^ between baseline and week 4 as proposed in [7]. For early significant hemoglobin decline analysis, patients for whom EPO therapy (N = 22) or RBV dose reduction (N = 4) was instituted before week 4 were excluded and 209 patients were included in this analysis.

For the SVR binary phenotype, all statistical analyses were conducted by means of logistic regression. Association with *IL28B* and *APOH* SNPs was tested by assuming an additive genetic model (i.e. the coding of the genotype represents the number of reference alleles 0, 1 or 2). We performed both univariate and multivariate analysis, including covariates previously identified as independent predictors of SVR in the CUPIC cohort (i.e.: prior treatment response, lead-in phase, platelet count and HCV-1 subtypes) [12]. Interaction between the *IL28B* SNP rs12979860 and binary covariates such as prior response to treatment (non-response versus relapse including breakthrough) and treatment group (TVR versus BOC), was modeled in the logistic regression framework by adding an interaction multiplicative term between the two main effects, e.g. *IL28B* SNP and the prior response to treatment.

The logistic regression framework was also used for the statistical analyses of the anemia related binary phenotypes (i.e. clinically relevant anemia and early significant hemoglobin decline). We performed both univariate and multivariate analysis, including *ITPA* SNPs (assuming an additive model), and other predictors of anemia (i.e.: age, sex, lead-in phase, hemoglobin at baseline, albumin at baseline <35g/L). To measure the joint effect of the two *ITPA* SNPs on anemia we considered a combined variable which estimates the severity of ITPA deficiency from rs1127354 and rs7270101 genotypes, as previously done in [7]. The severity of ITPA deficiency was defined as follows (S4 Table): Full ITPA activity (100%) was considered for rs1127354 C/C and rs7270101 A/A genotypes combination; 60% ITPA activity was considered for rs1127354 C/C and rs7270101 A/C genotypes combination; 30% ITPA activity was considered for rs1127354 C/C and rs7270101 C/C genotypes combination or rs1127354 A/C and rs7270101 A/A genotypes combination; and very low ITPA activity (0%) was considered for combined heterozygosity or rs1127354 A/A and rs7270101 A/A genotype combination [15]. Predicted ITPA activity was then considered as a quantitative covariate with four possible values (0; 0.3; 0.6; 1) in our logistic regression model. For the early hemoglobin decline phenotype, interaction between ITPA activity and the binary lead-in covariate was model in the logistic regression framework by adding a multiplicative interaction term between the two main effects, i.e. ITPA activity and lead-in.

All analyses were performed using the R software version 3.1.2 (http://cran.r-project), and p-values lower than 0.05 were considered as significant.

### Genotyping

We genotyped 10 SNPs (S1 Fig) using TaqMan SNP genotyping assays (Applied Biosystems Inc., Foster City, CA) a total of 10 SNPs, two *ITPA* SNPs (rs1127354 and rs7270101), two *IL28B* variants (rs12979860 and rs368234815) and six *APOH* SNPs (rs8178822 [16,17], rs12944940, rs10048158 [18], rs52797880, rs1801689 [16,19] and rs1801690 [16,20]) selected based on their potential impact on apoH plasma levels via a search on NCBI Pubmed and regulomeDB (score≥2b, http://regulomedb.org/).

## Results

### Patient characteristics

A total of 256 Caucasian patients were genotyped, 162 receiving TVR and 94 receiving BOC with comparable baseline characteristics (S1 Table). A total of 172 (67%) individuals were men. The mean (Standard deviation, SD) age at inclusion was 58.1y (9.8y). Prior treatment response was null in 31 (12%), partial in 108 (42%), breakthrough in 10 (4%) and relapse in 92 (36%) patients. As previously proposed [21,22], null and partial responders were grouped in a “prior non-response” category, and breakthrough and relapse were grouped in a “prior relapse” category for further analyses. Eighty six individuals (33%) were infected with genotype 1a, 147 (57%) with 1b and 21 (8%) with 1c. The mean (SD) hemoglobin level at baseline was 14.6g.dl^-1^ (1.7g.dl^-1^), and the mean (SD) platelet count at baseline was 150,000mm^-3^ (66,000mm^-3^). As expected by the protocol, patients treated with BOC were more likely to receive 4 weeks lead-in with PegIFN/RBV (95.7% vs 26.5%, P<0.001).

### Association of SVR status with APOH and IL28B SNPs

**Fig 1:**
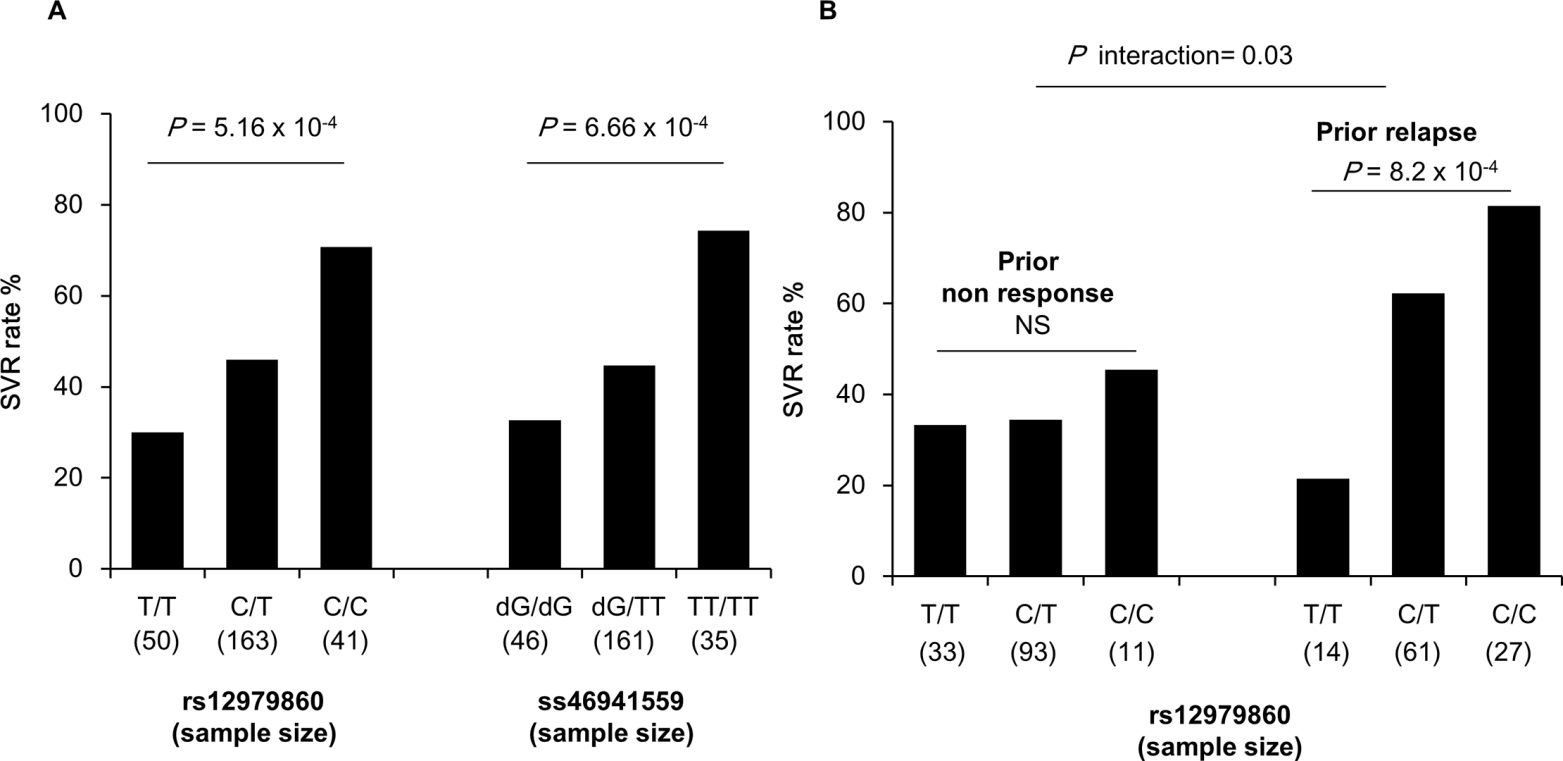
Rate of SVR by genotypes for *IL28B* SNPs. (A) SVR rates by genotypes for rs12979860 (left panel) and rs368234815 (right panel). (B) SVR rates by genotypes for rs12979860 according to prior treatment response.

SVR was achieved for 119 (46.5%) patients and no significant difference (P = 0.48) was observed between the two treatment groups. Results of univariate analysis of *IL28B* and *APOH* SNPs are presented in Table 1. All *APOH* SNPs were in Hardy Weinberg equilibrium (HWE), and some of them were in LD (S1 Fig). No significant association was observed between *APOH* SNPs and SVR. As previously observed in Caucasian individuals, *IL28B* variants, rs12979860 and rs368234815, were in almost complete LD (r² = 0.94; S1 Fig). As the results for both SNPs were very similar and the call rate of rs12979860 was slightly higher than that of rs368234815, results are presented only for rs12979860. As expected by the selection bias of the sample including only treatment-experienced patients, rs12979860 was not in HWE (*P* = 9.9x10^-6^) due to an enrichment of unfavorable allele T for clearance (52% in CUPIC patients vs 32% in the 1000 genomes European population (www.ensembl.org). Despite this skewed distribution, the favorable allele C of rs12979860 was significantly associated with SVR to triple therapy in univariate analysis (P = 2x10^-4^) with an Odds ratio (OR) of achieving SVR per increase of one copy of the favorable allele C (i.e. OR_C/CvsC/T_ or OR_C/TvsT/T_) of 2.35 (95% confidence interval: 1.50–3.70) (Table 1 and Fig 1A). The effect of rs12979860 genotype on SVR did not differ significantly between the two treatment groups (P_interaction_ = 0.3).

**Table 1:** Effects of *IL28B* and *APOH* variants on SVR and of *IPTA* variants on anemia in univariate analysis.

SNP	chr:position	Closest gene (variant type)	Reference/ alternative (aaf*)	n	call rate	HWE p-value	OR (95%CI)	*P-*value
**SVR phenotype**								
rs12979860	19:39248147	*IL28B* (intron)	C/T (0.52)	254	99.2	9.9 10^−6^	2.35 [1.50–3.70]	2.0x10^-4^
rs368234815	19:39248514	*IL28B* (splice)	TT/dG (0.52)	242	94.5	3.8 10^−7^	2.35 [1.46–3.79]	4.7x10^-4^
rs1801690	17:66212167	*APOH* (missense)	C/G (0.07)	251	98.0	0.62	1.47 [0.73–2.96]	0.28
rs1801689	17:66214462	*APOH* (missense)	A/C (0.04)	251	98.0	1	0.65 [0.26–1.61]	0.35
rs52797880	17:66220736	*APOH* (missense)	A/G (0.08)	242	94.5	1	1.40 [0.71–2.74]	0.33
rs8178822	17:66229411	*APOH* (5’UTR)	G/T (0.08)	253	98.8	1	1.31 [0.67–2.57]	0.43
rs12944940	17:66235598	*APOH* (intron)	T/C (0.21)	252	98.4	0.33	1.01 [0.64–1.57]	0.98
rs10048158	17:66240200	*APOH* (intron)	C/T (0.47)	251	98.0	0.42	0.96 [0.68–1.36]	0.84
**Clinically relevant anemia**								
rs1127354	20:3213196	*ITPA* (missense)	C/A (0.06)	255	99.6	1	1.36 [0.65–2.84]	0.42
rs7270101	20:3213247	*ITPA* (intron)	A/C (0.13)	255	99.6	0.83	1.31 [0.78–2.19]	0.31
**Early Hb decline**								
rs1127354	20:3213196	*ITPA* (missense)	C/A (0.06)	209	99.6	1	4.20 [1.38–12.8]	0.01
rs7270101	20:3213247	*ITPA* (intron)	A/C (0.13)	209	99.6	0.83	2.27 [1.20–4.29]	0.01

* aaf, alternative allele frequency.

We performed further multivariate analysis including covariates previously identified as independent predictors of SVR (i.e. prior treatment response, no lead-in phase, platelet count≥100,000mm^-3^ and HCV-1b subtype [12]) (S2 Table), and showed that rs12979860 was independently associated with SVR (OR = 2.05[1.24–3.48], p = 5.9x10^-3^). The best predictor of SVR in the multivariate analysis was previous relapse to PegIFN/RBV (OR = 2.69[1.50–4.88], p = 9.6x10^-4^). Hence, we further explored the combined effect of rs12979860 and prior treatment response on SVR, and found a significant interaction (p = 0.03) between these two factors. As shown in Fig 1B, the effect of rs12979860 on SVR was observed only in prior relapse (P = 8.2x10^-4^) with a stronger OR of 3.80[1.82–8.92], while this effect is no more significant (P = 0.6) in prior non-responders with an OR of 1.20[0.63–2.33]. Multivariate analysis restricted to previous relapse patients (S2 Table) showed that rs1279860 has the most significant effect on SVR among other known predictors (P = 4.4x10^-4^) with an OR of 5.01[2.16–13.3]. Overall, our results suggest that the effect of rs12979860 on triple therapy-induced clearance in treatment-experienced patients is restricted to those who experienced prior PegIFN/RBV relapse.

### Association of anemia with ITPA SNPs

**Fig 2:**
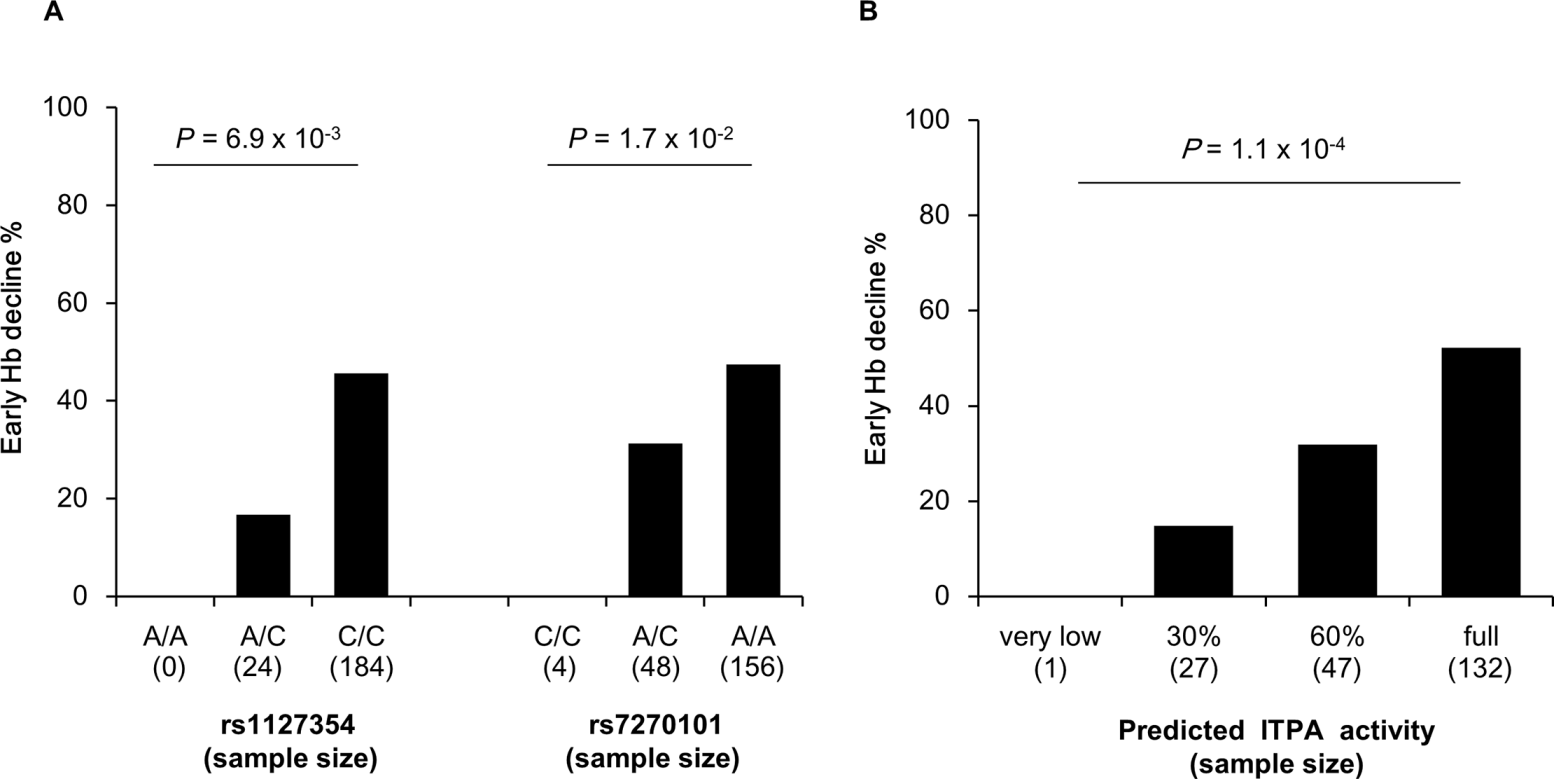
Rate of early hemoglobin decline by genotypes for rs1127354 and rs7270101 (A) and by predicted ITPA activity (B). Severity of ITPA deficiency was defined as in *Fellay et al* [7] considering rs1127354 C and rs7270101 A equivalent low activity variants.

Clinically relevant anemia and early significant Hb decline were observed in 155 (60.5%) and 89 (42.6%) patients, respectively. Both *ITPA* SNPs were in HWE (Table 1), and were not in LD (S1 Fig). The frequencies of major alleles of rs1127354 (C) and rs7270101 (A), associated with normal ITPA activity, were 0.94 and 0.87, respectively. In univariate analysis, clinically relevant anemia was associated neither with rs1127354 (p = 0.42), nor with rs7270101 (p = 0.31). In contrast, early significant Hb decline was significantly associated with rs1127354 (OR_C/CvsC/A_ = OR_C/AvsA/A_ = 4.20[1.38–12.8], p = 0.01), and with rs7270101 (OR_A/AvsA/C_ = OR_A/CvsC/C_ = 2.27[1.20–4.29], P = 0.01) (Table 1 and Fig 2A). In multivariate analysis including other predictors of anemia (i.e. age in years, sex, lead-in, Hb at baseline and albumin at baseline [10,12]), rs1127654 and rs7270101 major alleles were strongly and independently associated with early significant Hb decline (rs1127354, OR = 7.83[2.64–29.2], *P* = 6.0x10^-4^; rs7270101, OR = 3.28[1.65–6.95], *P* = 1.2x10^-3^).

We further explored the joint effect of rs1127354 and rs7270101 by considering as a quantitative covariate the severity of ITPA deficiency estimated from the genotypes at rs1127354 and rs7270101, as shown in S4 Table [7]. Consistent with the SNP effects, clinically relevant anemia was not associated (p = 0.12) with ITPA activity while there was a strong effect of ITPA activity (P = 1.1x10^-4^) on the rate of early Hb decline ranging from 15% for 30% activity to 52.3% for full activity (Fig 2B). Early significant Hb decline was defined at week 4 of therapy and was the consequence of either PegIFN/RBV alone for patients having had a lead-in or PegIFN/RBV plus TVR or BOC for individuals who started triple therapy without a lead-in. As already shown [8,9], the absence of lead-in (and consequently the triple therapy) was significantly associated (P = 6.4x10^-3^ in multivariate analysis) with early Hb decline (S3 Table). However, refined analysis showed that the effect of ITPA activity on early Hb decline did not differ significantly (P_interaction_ = 0.68) between the groups of patients with or without lead-in, with similar OR values observed in these two groups of 2.45[1.28–5.47] and 2.99[1.62–5.97], respectively. These results indicate that PIs increase the risk of early Hb decline, but do not have a significant influence on the relationship between early Hb decline and ITPA activity.

## Discussion

Triple therapy using BOC or TVR remains the reference treatment for chronically HCV-1 infected patients in a large number of countries, and is of major importance for patients with cirrhosis who are at risk to develop severe complications as liver failure or hepatocellular carcinoma [23]. However, the overall chance of success with triple therapy is around 50% in those cirrhotic patients and the risk of adverse effects remains high [12]. Therefore, providing information that could help to the prediction of achieving SVR for cirrhotic patients under triple therapy is of major interest [23]. In this study, the unique CUPIC cohort of well characterized treatment experienced cirrhotic patients allowed us refining the association between *IL28B* SNPs and SVR to triple therapy, and between *ITPA* SNPs and anemia. *IL28B* genotype is the strongest predictor of SVR to the standard PegIFN/RBV therapy in patients chronically infected with HCV-1. In treatment-naïve HCV-1 patients receiving TVR or BOC in combination with PegIFN/RBV, the association between *IL28B* genotype and SVR remains clinically relevant [21,23,24]. We report here that *IL28B* alleles favorable for clearance were associated with a twofold increase of SVR rate in a cohort of treatment-experienced patients of Caucasian origin, chronically infected by HCV-1, with compensated cirrhosis and receiving either TVR or BOC in triple therapy. Refined analysis showed that the effect of *IL28B* SNPs was restricted to individuals who previously relapsed (i.e. breakthrough or relapse) to PegIFN alone or PegIFN/RBV therapy, with a stronger effect of these SNPs on SVR in this population. This result suggests that TVR and BOC may potentiate *IL28B*-dependent clearance transiently induced by PegIFN/RBV therapy, and that *IL28B*-independent mechanisms are involved in the non-response to PegIFN/RBV therapy.

Several studies including both treatment-naïve and treatment experienced HCV patients receiving TVR- or BOC-based therapy have also consistently identified *IL28B* genotype as a predictor of SVR independent of treatment history [25–28]. However, results of the few studies focusing only on treatment-experienced patients were less conclusive. In a Japanese cohort of 103 treatment-experienced patients, mono-infected with HCV-1b and receiving TVR, the *IL28B* variant, rs8099917, was an independent predictor of SVR [29]. In the RESPOND-2 study of BOC-based therapy in treatment-experienced patients (N = 207), rs12979860 C/C genotype was predictive of a good interferon response at week 4 but only a non-statistically significant trend was observed with SVR [21]. In the REALIZE study of TVR-based therapy in 422 treatment-experienced patients, SVR rates were slightly higher among patients with rs12979860 C/C genotype compared with C/T and T/T genotypes but the difference was not statistically significant [22]. In these two latter studies, patients included variable proportions of prior responders (slightly higher in the RESPOND-2 than in the REALIZE study), and a rather low proportion of cirrhotic patients (<30%). Further studies are needed to identify the factors, like prior response, ethnic origin, liver fibrosis status, or HCV-1 genotype, which could explain the differences observed in the strength of association between SVR and *IL28B* genotype in treatment-experienced patients receiving either TVR or BOC.

No significant association was observed between *APOH* SNPs and SVR. One explanation could be that the impact of each individual variant on apoH levels is not large enough to further impact on SVR. Moreover, our study was underpowered to detect an association signal between SVR and variant with minor allele frequency below 0.1, which is the case for four of the six tested *APOH* SNPs. As an example, the power to detect an association between SVR and the missense SNP rs1801690 was 55% at the 0.05 type I error level for an additive OR of 2. Finally, despite a careful literature search and public database screening, we may have missed some variants impacting on apoH levels that are not identified yet.

Anemia is a well-established adverse event with PegIFN/RBV treatment of chronic HCV infection and the addition of PIs, such as TVR and BOC, has significantly increased its incidence [8,9]. Our data are consistent with this observation as the patients without lead-in had a higher rate of early Hb decline. The role of ITPA polymorphisms on early Hb decline and/or early anemia was identified by GWAS [7] and further replicated in different ethnic groups treated both by PegIFN/RBV [30–35], and by a triple combination therapy with TVR [31,36]. Here, we confirmed the protective effect of low ITPA activity variants on early Hb decline in treatment-experienced patients having received pegIFN/RBV alone or triple therapy combination for 4 weeks. We could also show that the relationship between *ITPA* SNPs and early Hb decline was not influenced by the presence of PIs, indicating that the effect of PIs on Hb decline is probably independent of ITPA activity. In our study, ITPA deficiency did not protect against clinically relevant anemia, which has a broader definition based on both Hb decline and anemia management during the whole period, a finding consistent with the previous study of Aghemo et al. [36].

Overall, our results suggest that the screening of rs12979860 remains interesting for decision making in Caucasian difficult-to-treat HCV-1 patients (in particular if they presented a prior PegIFN/RBV relapse) with compensated cirrhosis eligible for a PI-based triple therapy. In those patients, the genotyping of ITPA SNPs are very useful to predict the development of early severe Hb decline.

## Supporting Information

S1 Fig LD plot of *APOH*, *IL28B* and *ITPA* SNPs (chromosome 17, 19 and 20 respectively). (DOCX)

S1 Table Characteristics of the patients (DOCX)

S2 Table Factors related to SVR: multivariate analysis. (DOCX)

S3 Table Factors related to early hemoglobin decline: univariate and multivariate analysis. (DOCX)

S4 Table Predicted ITPA activity according to genotypes at the two ITPA SNPs and corresponding number of observed patients for early hemoglobin decline analysis. (DOCX)
