# Supplementary material for: Impact of IL28B, APOH and ITPA Polymorphisms on Efficacy and Safety of TVR- or BOC-Based Triple Therapy in Treatment-Experienced HCV-1 Patients with Compensated Cirrhosis from the ANRS CO20-CUPIC Study
Source: PLoS One. 2015 Dec 15;10(12):e0145105. doi: 10.1371/journal.pone.0145105 (PMC4682920; doi:10.1371/journal.pone.0145105)

**S1 Figure. LD plot of *APOH*, *IL28B* and *ITPA* SNPs (chromosome 17, 19 and 20 respectively).**

Pairwise LD is measured by the r² value. *APOH* SNPs were selected based on their potential impact on apoH plasma levels via a search on NCBI Pubmed and regulomeDB (score≥2b, <http://regulomedb.org/>): rs8178822, rs12944940, rs10048158, rs52797880, rs1801689 and rs1801690.


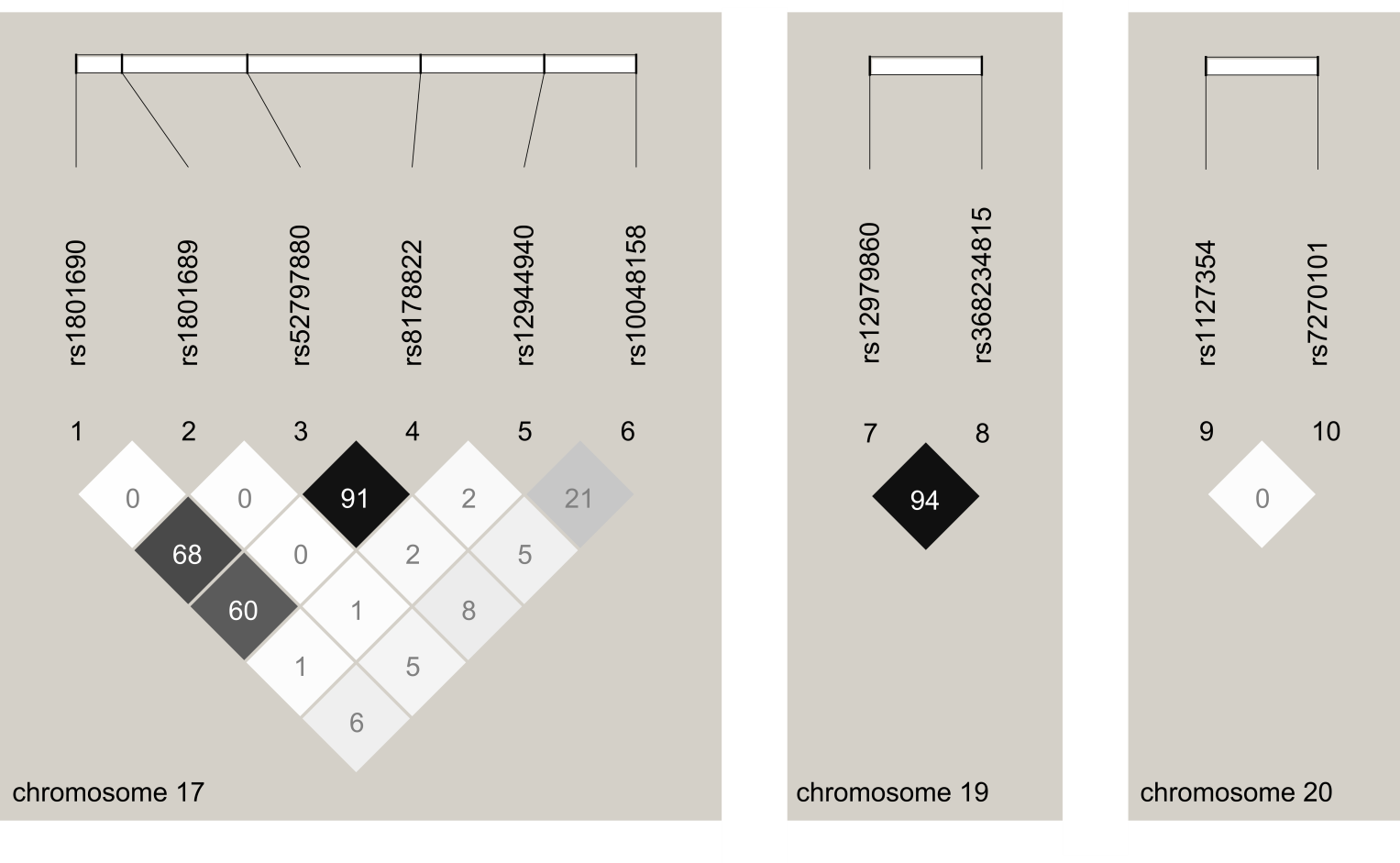

Supplement: S1 Fig — (DOCX) [file pone.0145105.s001.docx]
