# Supplementary material for: Impact of IL28B, APOH and ITPA Polymorphisms on Efficacy and Safety of TVR- or BOC-Based Triple Therapy in Treatment-Experienced HCV-1 Patients with Compensated Cirrhosis from the ANRS CO20-CUPIC Study
Source: PLoS One. 2015 Dec 15;10(12):e0145105. doi: 10.1371/journal.pone.0145105 (PMC4682920; doi:10.1371/journal.pone.0145105)
Supplement: S1 Table — (DOCX) [file pone.0145105.s002.docx]

**S1 Table. Characteristics of the patients**

| Characteristics | | Telaprevir n=162 | | Boceprevir n=94 | | All n=256 | | *P* value * | |
| --- | --- | --- | --- | --- | --- | --- | --- | --- | --- |
| Mean age (range), yr | | 58.7 (30-83) | | 57.1 (34-79) | | 58.1 (30-83) | | 0.22 | |
| Male sex, n (%) | | 111 (68.6) | | 61 (64.9) | | 172 (67.2) | | 0.55 | |
| Mean body mass index, (±SD) kg/m² | | 26.3 (±3.9) | | 25.5 (±4.0) | | 26.0 (±3.9) | | 0.13 | |
| Treatment history, n (%) | |  | |  | |  | | 0.80 | |
| Prior relapse | | 57 (35.2) | | 35 (37.2) | | 92 (35.9) | |  | |
| Breakthrough | | 5 (3.1) | | 5 (5.3) | | 10 (3.9) | |  | |
| Prior partial response | | 68 (42.0) | | 40 (42.6) | | 108 (42.2) | |  | |
| Prior null response | | 22 (13.5) | | 9 (9.6) | | 31 (12.1) | |  | |
| Undetermined | | 10 (6.2) | | 5 (5.3) | | 15 (5.9) | |  | |
| Mean hemoglobin level (range), g/dL | | 14.6 (10.1-19.7) | | 14.6 (9.1-18.4) | | 14.6 (9.1-19.7) | | 0.93 | |
| Mean neutrophil count (range), 10^9^/mm^3^ | | 3.3 (0.9-12.2) | | 3.4 (0.5-8.5) | | 3.3 (0.5-12.2) | | 0.76 | |
| Mean platelet count (range), /mm^3^ | | 150 (18-387) | | 149 (39-346) | | 150 (18-387) | | 0.92 | |
| Mean ALT (range), IU/L | | 91 (11-456) | | 101 (20-460) | | 95 (11-460) | | 0.28 | |
| Mean prothrombin time ratio (range), % | | 87 (27-100) | | 88 (27-100) | | 88 (27-100) | | 0.81 | |
| Mean creatinine level (range), µmol/L | | 69 (35-112) | | 67 (42-109) | | 68 (35-112) | | 0.13 | |
| HCV genotype 1 subtype, n (%) | |  | |  | |  | | 0.33 | |
| 1a | | 53 (32.7) | | 33 (35.1) | | 86 (33.6) | |  | |
| 1b | | 96 (59.3) | | 51 (54.3) | | 147 (57.4) | |  | |
| 1c | | 13 (8.0) | | 8 (8.5) | | 21 (8.2) | |  | |
| Undetermined | | - | | 2 (2.1) | | 2 (0.8) | |  | |
| Lead-in, n(%) | | 43 (26.5) | | 90 (95.7) | | 133 (52.0) | | < 0.001 | |
| SVR, n(%) | | 78 (48.1) | | 41 (43.6) | | 119 (46.5) | | 0.48 | |
| Clinically relevant anemia, n(%) | | 94 (58.1) | | 61 (64.9) | | 155 (60.5) | | 0.24 | |
| Early hemoglobin decline, n(%) | | 62 (48.1) | | 27 (33.8) | | 89 (42.6) | | 0.09 | |

* Chi-square for binary variable and Student t test for quantitative variable (telaprevir vs boceprevir).

Lead-in consisted of 4 weeks of PegIFN/RBV therapy before the introduction of either telaprevir or boceprevir.

Sustained virological response (SVR) was measured 12 weeks after the end of therapy. Clinically relevant anemia was defined by grade 2, 3 or 4 anemias (i.e. Hemoglobin [Hb] < 9.5 g/dL) and/or blood transfusion and/or use of EPO. Early hemoglobin decline was defined as a decrease of hemoglobin of at least 3 points between baseline and week 4.

Complete data for 256 patients except for body mass index (n=249), hemoglobin level (n=251), neutrophil count (n=250), platelet count (n=250), ALT (n=250), prothrombin time (n=224), creatinine level (n=245) and early hemoglobin decline (n=209).
