# Supplementary material for: Impact of IL28B, APOH and ITPA Polymorphisms on Efficacy and Safety of TVR- or BOC-Based Triple Therapy in Treatment-Experienced HCV-1 Patients with Compensated Cirrhosis from the ANRS CO20-CUPIC Study
Source: PLoS One. 2015 Dec 15;10(12):e0145105. doi: 10.1371/journal.pone.0145105 (PMC4682920; doi:10.1371/journal.pone.0145105)
Supplement: S2 Table — (DOCX) [file pone.0145105.s003.docx]

**S2 Table. Factors related to SVR: multivariate analysis**

|  | | All  N=233 | | |  | Prior relapse  N=101 | | |  | Prior non response  N=132 | | |
| --- | --- | --- | --- | --- | --- | --- | --- | --- | --- | --- | --- | --- |
|  | OR | | 95% CI | *P* value |  | OR | 95% CI | *P* value |  | OR | 95% CI | *P* value |
| Age ≥ 65y | 0.65 | | [0.32-1.29] | 0.22 |  | 0.22 | [0.06-0.72] | 0.02 |  | 0.85 | [0.33-2.16] | 0.74 |
| Female sex | 0.78 | | [0.41-1.45] | 0.43 |  | 1.26 | [0.45-3.70] | 0.66 |  | 0.66 | [0.27-1.51] | 0.33 |
| Prior treatment response |  | |  |  |  |  |  |  |  |  |  |  |
| Non response | 1 | |  |  |  | - |  |  |  | - |  |  |
| Relapse | 2.69 | | [1.50-4.88] | 9.6 10^-4^ |  | - |  |  |  | - |  |  |
| No lead-in phase | 1.63 | | [0.75-3.60] | 0.21 |  | 6.14 | [1.51-28.1] | 0.01 |  | 0.97 | [0.36-2.72] | 0.96 |
| Boceprevir vs Telaprevir | 1.14 | | [0.52-2.53] | 0.74 |  | 0.66 | [0.17-2.42] | 0.52 |  | 1.67 | [0.60-4.78] | 0.33 |
| Platelet count≥100,000/mm3 | 2.61 | | [1.30-5.43] | 8.3 10^-3^ |  | 4.69 | [1.37-18.1] | 0.02 |  | 2.60 | [1.02-7.32] | 0.054 |
| HCV subtype |  | |  |  |  |  |  |  |  |  |  |  |
| 1a + 1c | 1 | |  |  |  | 1 |  |  |  | 1 |  |  |
| 1b | 2.62 | | [1.44-4.86] | 1.8 10^-3^ |  | 2.47 | [0.91-7.11] | 0.08 |  | 2.90 | [1.28-6.86] | 0.01 |
| rs12979860 | 2.05 | | [1.24-3.48] | 5.9 10^-3^ |  | 5.01 | [2.16-13.3] | 4.4 10^-4^ |  | 1.17 | [0.59-2.35] | 0.64 |
