# Supplementary material for: Impact of IL28B, APOH and ITPA Polymorphisms on Efficacy and Safety of TVR- or BOC-Based Triple Therapy in Treatment-Experienced HCV-1 Patients with Compensated Cirrhosis from the ANRS CO20-CUPIC Study
Source: PLoS One. 2015 Dec 15;10(12):e0145105. doi: 10.1371/journal.pone.0145105 (PMC4682920; doi:10.1371/journal.pone.0145105)
Supplement: S3 Table — (DOCX) [file pone.0145105.s004.docx]

**S3 Table. Factors related to early hemoglobin decline: univariate and multivariate analysis**

|  | Univariate analysis (N=209) | | |  | Multivariate analysis (N=207) | | |
| --- | --- | --- | --- | --- | --- | --- | --- |
|  | OR | 95% CI | *P* value |  | OR | 95% CI | *P* value |
| Age, y | 1.02 | [0.99-1.05] | 0.22 |  |  |  |  |
| Female sex | 1.24 | [0.69-2.23] | 0.48 |  |  |  |  |
| No lead-in phase | 2.14 | [1.22-3.74] | 7.7x10^-4^ |  | 2.65 | [1.43-5.05] | 2.4x10^-3^ |
| Hemoglobin at baseline* | 0.07 | [0.01-0.57] | 0.01 |  | 0.06 | [0.003-0.30] | 6.4x10^-3^ |
| Albumin at baseline < 35g/L | 0.99 | [0.38-2.59] | 0.98 |  |  |  |  |
| rs1127354 | 4.20 | [1.38-12.8] | 0.01 |  | 7.83 | [2.64-29.2] | 6.0x10^-4^ |
| rs7270101 | 2.27 | [1.20-4.29] | 0.01 |  | 3.28 | [1.65-6.95] | 1.2x10^-3^ |

Early Hb decline was defined by a decrease of hemoglobin of at least 3 points between baseline and week 4.

Lead-in consisted of 4 weeks of PegIFN/RBV therapy before the introduction of either telaprevir or boceprevir.

*Baseline hemoglobin level of 12g/dL or lower for women and 13g/dl or lower for men.
