# Supplementary material for: Impact of IL28B, APOH and ITPA Polymorphisms on Efficacy and Safety of TVR- or BOC-Based Triple Therapy in Treatment-Experienced HCV-1 Patients with Compensated Cirrhosis from the ANRS CO20-CUPIC Study
Source: PLoS One. 2015 Dec 15;10(12):e0145105. doi: 10.1371/journal.pone.0145105 (PMC4682920; doi:10.1371/journal.pone.0145105)
Supplement: S4 Table — (DOCX) [file pone.0145105.s005.docx]

**S4 Table. Predicted ITPA activity according to genotypes at the two ITPA SNPs and corresponding number of observed patients for early hemoglobin decline analysis**

|  |  | **rs1127354** | | |  |
| --- | --- | --- | --- | --- | --- |
|  | **A/A** | **A/C** | **C/C** | **Total** |  |
| **rs7270101** | **C/C** | NA (n=0) | NA (n=0) | 30% (n=4) | 4 |
|  | **A/C** | NA (n=0) | Very low (n=1) | 60% (n=47) | 48 |
|  | **A/A** | Very low (n=0) | 30% (n=23) | 100% (n=132) | 155 |
|  | **Total** | 0 | 24 | 183 | 207 |
